# Supplementary material for: Influence of Speech and Cognitive Load on Balance and Timed up and Go
Source: Brain Sci. 2022 Jul 31;12(8):1018. doi: 10.3390/brainsci12081018 (PMC9405849; doi:10.3390/brainsci12081018)
Supplement: Supplementary file 1 [file brainsci-12-01018-s001.zip › brainsci-1796289-supplementary.pdf]

Supplementary Table S1: Difference [95% CI] and *p*-value of group comparisons after post-hoc corrections

Variable	Conditions	Control	Oral 3	Mental 3	Oral 7
MVml (mm/s)	Oral 3	1.11 [−0.38 ; 2.60]<br>P = 0.243			
	Mental 3	0.27 [−1.24 ; 1.79]<br>P = 0.987	0.84 [−0.66 ; 2.34]<br>p = 0.533		
	Oral 7	**1.75 [0.26 ; 3.24]**<br>**p = 0.012**	0.63 [−0.84 ; 2.11]<br>P = 0.760	**1.47 [0.03 ; 3.01]**<br>**p = 0.047**	
	Mental 7	0.27 [−1.22 ; 1.76]<br>P = 0.987	−0.84 [−2.32 ; 0.63]<br>P = 0.514	−0.01 [−1.50 ; 1.50]<br>P = 0.997	**1.48 [0.02 : 3.00]**<br>**p = 0.049**
MVap (mm/s)	Oral 3	**1.66 [0.20 ; 3.12]**<br>**P = 0.003**			
	Mental 3	**−0.11 [−1.46 ; 1.24]**<br>**P = 0.971**	**1.77 [0.36 ; 3.28]**<br>**P = 0.003**		
	Oral 7	**1.80 [0.39 ; 3.21]**<br>**P = 0.002**	0.14 [−1.41 ; 1.70]<br>P = 0.917	**1.91 [0.45 ; 3.38]**<br>**P = 0.002**	
	Mental 7	−0.10 [1.40 ; 1.19]<br>P = 0.917	**−1.76 [−3.22 ; −0.30]**<br>**P = 0.003**	0.01 [−1.34 ; 1.36]<br>P = 0.985	**1.91 [0.49 ; 3.32]**<br>**P = 0.001**
TMV (mm/s)	Oral 3	**1.69 [0.18 ; 3.21]**<br>**P = 0.024**			
	Mental 3	−0.15 [−1.56 ; 1.25]<br>P = 0.998	**1.86 [0.29 ; 3.42]**<br>**P = 0.014**		
	Oral 7	**1.79 [0.32 ; 3.25]**<br>**P = 0.010**	0.09 [−1.53 ; 1.71]<br>P = 0.999	**1.94 [0.42 ; 3.47]**<br>**P = 0.006**	
	Mental 7	−0.17 [−1.51 ; 1.18]<br>P = 0.997	**−1.86 [−3.38 ; −0.35]**<br>**P = 0.009**	−0.01 [−1.41 ; 1.39]<br>P = 0.998	**1.95 [0.49 ; 3.42]**<br>**P = 0.003**
TUG (s)	Oral 3	**0.94 [0.15 ; 1.74]**<br>**P = 0.010**			
	Mental 3	**0.84 [0.05 ; 1.62]**<br>**P = 0.032**	0.11 [−0.68 ; 0.90]<br>P = 0.995		
	Oral 7	**1.42 [0.64 ; 2.22]**<br>**$P = 1.6\times10^{-5}$**	0.48 [−0.31 ; 1.27]<br>P = 0.449	0.59 [−0.20 ; 1.38]<br>P = 0.240	
	Mental 7	**0.97 [0.18 ; 1.76]**<br>**P = 0.007**	0.03 [−0.76 ; 0.82]<br>P = 0.999	0.13 [−0.65 ; 0.92]<br>P = 0.989	0.45 [−0.33 ; 1.24]<br>P = 0.507

MVml; mean medio-lateral velocity, MVap; mean antero-posterior velocity, TMV; Total Mean Velocity
